# Effectiveness of Amniotic Membrane Transplantation in Corneal Ulcer Healing: A Systematic Review

**DOI:** 10.7759/cureus.86231

**Published:** 2025-06-17

**Authors:** Anas E Ahmed, Eyad M Albarrati, Abdulaziz Y Muyidi, Yara M Adawi, Hani A Al-Ghamdi, Ahmed A Alahmadi, Abdulrahman F Alshehri, Faisal H Alfaify, Sultan M Almugharrid, Ali S Alkebiri, Abdulmajeed A Alghamdi, Faisal A Alsalouli, Hussam A Almalki, Abdullah M Alsharif, Rayan S Almugharrid

**Affiliations:** 1 Community Medicine, Jazan University, Jazan, SAU; 2 College of Medicine, Jazan University, Jazan, SAU; 3 College of Medicine, Al-Baha University, Al-Baha, SAU; 4 College of Medicine, Umm Al-Qura University, Mecca, SAU; 5 Medicine and Surgery, Umm Al-Qura University, Mecca, SAU; 6 College of Medicine, University of Bisha, Bisha, SAU; 7 College of Medicine, King Khalid University, Abha, SAU

**Keywords:** amniotic membrane transplantation, corneal ulcer, epithelial healing, eye inflammation, graft failure, neurotrophic keratitis, ocular surface, regenerative therapy, systematic review, visual acuity

## Abstract

Corneal ulcers are a major cause of visual morbidity and blindness worldwide, and conventional treatments often fall short, particularly in chronic or neurotrophic cases. Amniotic membrane transplantation (AMT) has emerged as a promising adjunctive therapy due to its regenerative, anti-inflammatory, and anti-scarring properties. This systematic review evaluated the effectiveness of AMT in promoting corneal ulcer healing by assessing epithelialization time, visual acuity improvement, and complications. A comprehensive search of five databases - PubMed, Cochrane Library, Scopus, Web of Science, and the Virtual Health Library - was conducted up to April 20, 2024, including randomized controlled trials and prospective clinical studies published in English over the past decade. Nine studies were included, involving diverse patient populations and ulcer etiologies. AMT consistently accelerated epithelial healing, with reduced healing times compared to controls, early postoperative pain relief, and visual acuity improvements, particularly in central ulcers. Adverse effects were infrequent, and graft failure was rare. However, differences in technique and outcome measures limited comparability across studies. Overall, AMT appears to be a safe and effective adjunctive treatment for corneal ulcers, enhancing healing, comfort, and vision. Further validation through standardized protocols and high-quality multicenter trials is recommended to support clinical implementation.

## Introduction and background

Corneal ulcers represent a serious ophthalmic condition characterized by the loss of corneal epithelium with stromal involvement, often accompanied by inflammation, edema, and pain. Globally, they are a leading cause of visual impairment and blindness, particularly in developing countries, where access to prompt ophthalmologic care may be limited [1]. Etiologies are diverse and include microbial infections (bacterial, fungal, viral, or protozoal), trauma, neurotrophic keratopathy, exposure keratopathy, contact lens wear, and immune-mediated diseases such as Stevens-Johnson syndrome and ocular cicatricial pemphigoid [2].

Standard management typically includes antimicrobial therapy for infectious ulcers, lubricants, and protective measures, such as bandage contact lenses or tarsorrhaphy, for exposure-related ulcers, and supportive treatment for neurotrophic or non-healing defects [1,2]. However, in many chronic or refractory cases, especially those associated with underlying neurotrophic or autoimmune pathologies, traditional therapies are often insufficient to achieve full re-epithelialization or prevent stromal degradation [3].

Amniotic membrane transplantation (AMT) has emerged as a promising adjunctive therapy in such cases. The amniotic membrane is the innermost layer of the placenta, composed of a basement membrane and an avascular stromal matrix rich in growth factors, cytokines, and extracellular matrix proteins. These components contribute to its anti-inflammatory, anti-fibrotic, anti-angiogenic, and pro-epithelialization properties [3,4]. Additionally, it acts as a biological scaffold, providing mechanical support and promoting epithelial cell migration, adhesion, and differentiation [2].

AMT has been successfully used in a wide array of ocular surface disorders, including persistent epithelial defects, chemical and thermal burns, pterygium surgery, and limbal stem cell deficiency [3]. Its role in treating corneal ulcers, particularly in refractory, non-infectious, or neurotrophic cases, has been increasingly explored in recent years. However, published studies have shown variable results, often influenced by differences in surgical technique (e.g., single-layer vs. multilayer grafting), type of amniotic membrane (cryopreserved vs. lyophilized), ulcer etiology, and outcome measures [1-4].

Therefore, a comprehensive synthesis of the available clinical evidence is warranted. This systematic review aims to evaluate the effectiveness of AMT in the management of corneal ulcers, focusing on its impact on epithelial healing time, visual acuity improvement, recurrence, and complication rates. By analyzing data from prospective clinical studies and randomized controlled trials, we aim to provide evidence-based guidance for the role of AMT in corneal ulcer therapy.

## Review

Methods

Literature Search Strategy

This systematic review was conducted following the Preferred Reporting Items for Systematic Reviews and Meta-Analyses (PRISMA) guidelines. A comprehensive search was performed across five major biomedical databases, PubMed, Cochrane Central Register of Controlled Trials (CENTRAL), the Virtual Health Library, Scopus, and Web of Science, to identify relevant studies published up to April 20, 2024. The search combined Medical Subject Headings (MeSH) and free-text keywords, using terms such as "amniotic membrane" OR "AMT" AND "corneal ulcer," adapted to each database’s indexing system. Language restriction to English was applied. To ensure relevance and quality, only studies involving human subjects with corneal ulcers treated with amniotic membrane transplantation were included, covering randomized controlled trials and observational designs.

Eligibility Criteria

The eligibility criteria were defined using the PICO (Population, Intervention, Comparison, Outcome, Study Design) framework. Included studies met the following conditions: randomized controlled trials, prospective comparative studies, or observational case series published in English within the last 10 years; enrolled patients of any age or sex diagnosed with corneal ulcers or persistent epithelial defects from various causes, such as neurotrophic, infectious, or exposure-related; evaluated amniotic membrane transplantation as a treatment modality; compared AMT to conventional treatments, including bandage contact lenses, conjunctival flaps, or medical therapy alone where applicable; and reported clinical outcomes such as epithelial healing rate and time, visual acuity changes, pain relief, recurrence, or complications. Studies involving non-human subjects, in vitro research, lacking full-text access, or non-original articles (reviews, editorials, case reports, original articles, conference abstracts, or trial registrations) were excluded.

Study Selection

Two independent reviewers screened titles and abstracts of all retrieved records according to the predefined criteria. Full-text articles were then assessed for eligibility. Discrepancies regarding study inclusion were resolved through discussion, and, if necessary, a third reviewer was consulted to ensure consistency and objectivity.

Data Extraction

Data extraction was performed in detail using a standardized form. Extracted information included study design, country, sample size, participant demographics (age, gender), corneal ulcer etiology (infectious, neurotrophic, exposure-related), intervention specifics (type of AMT, number of layers, surgical technique), comparator treatments, follow-up duration, and primary outcomes, such as epithelial healing rate and time, visual acuity changes, pain relief, recurrence, and complications. Any disagreements in data extraction were resolved by consensus or with input from a third reviewer.

Quality Assessment

Methodological quality was independently assessed by two reviewers using the Modified Downs and Black checklist, appropriate for both randomized and non-randomized studies, without any further changes to the checklist [5]. The checklist evaluates five domains: reporting, external validity, internal validity (bias), internal validity (confounding), and statistical power. Based on total scores, studies were classified as excellent (26-28), good (20-25), fair (15-19), or poor (≤14). Any scoring disagreements were resolved through discussion until a consensus was reached.

Results

Study Selection

**Figure 1:**
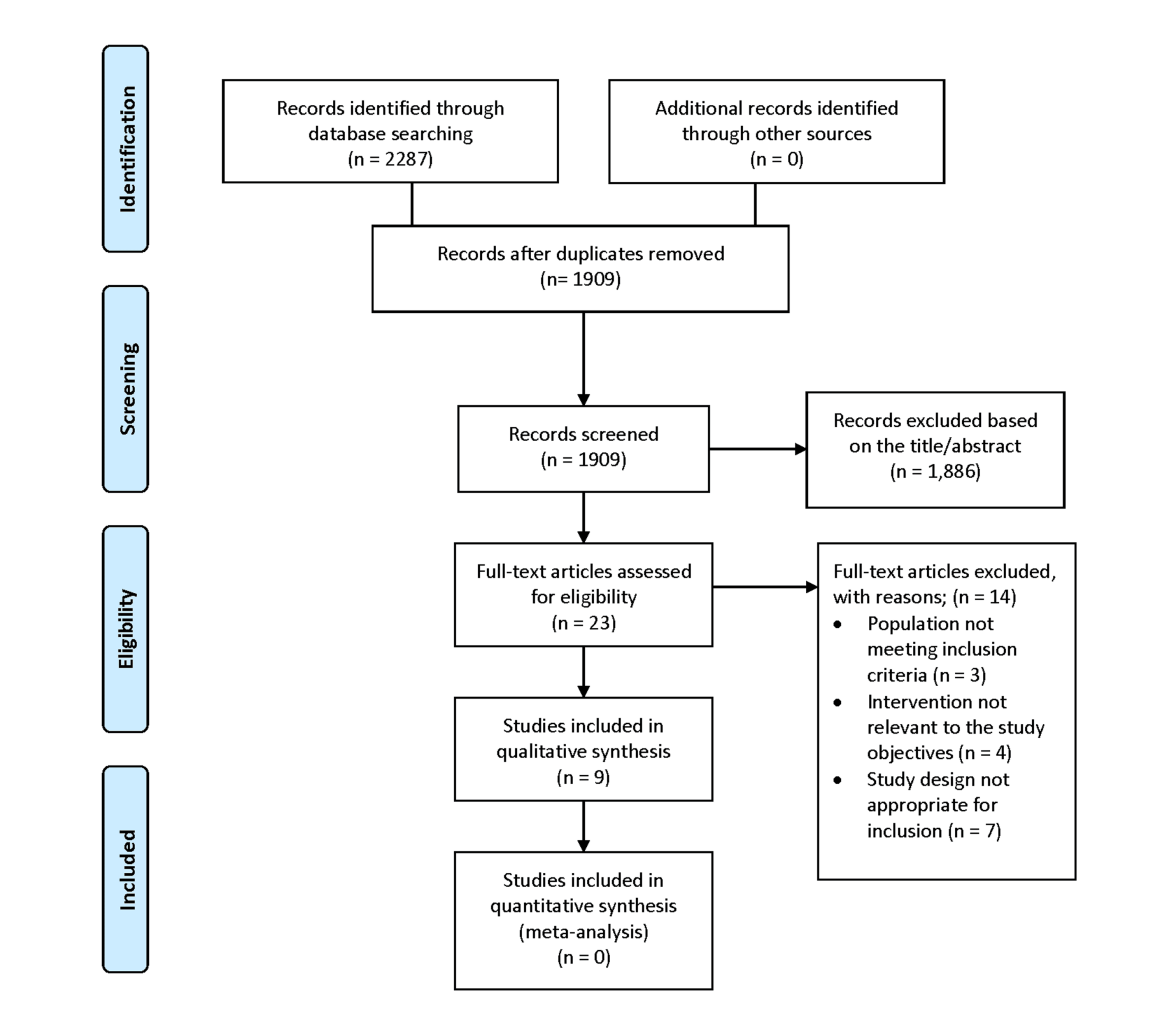
PRISMA flowchart of studies search and selection PRISMA: Preferred Reporting Items for Systematic Reviews and Meta-Analyses

The initial database search identified 2,287 records: PubMed (n = 262), Cochrane Library (n = 29), Virtual Health Library (n = 324), Web of Science (n = 687), and Scopus (n = 985), using the search terms ("amniotic membrane" OR "AMT") AND "corneal ulcer". After removing 378 duplicates, 1,909 unique records were screened. Title and abstract screening excluded 1,886 articles for irrelevance. Twenty-three full-text articles were assessed, with 14 excluded for not meeting inclusion criteria: 3 for irrelevant populations, 4 for inappropriate interventions, and 7 for unsuitable study designs. Nine studies were included in the qualitative synthesis [1,6-13]. No studies qualified for quantitative meta-analysis (Figure 1).

Study Characteristics

The 9 included studies, published between 1997 and 2024, involved a total of 322 eyes with persistent corneal epithelial defects or ulcers treated with amniotic membrane transplantation (AMT). Study designs comprised randomized controlled trials [1,8,12], prospective comparative studies [10,11], and observational case series [6,7,9,13]. Sample sizes ranged from 7 to 100 eyes. Most studies enrolled adults with mean ages ranging from 25.1 to 69.9 years. Gender distribution was generally balanced (Table 1).

**Table 1:** Studies evaluating amniotic membrane transplantation for corneal ulcers and persistent epithelial defects This table summarizes prospective and randomized studies examining the use of amniotic membrane transplantation in the management of corneal ulcers and persistent epithelial defects. Interventions varied in technique and layering of the cryopreserved amniotic membrane, with some studies including control or comparator arms. Key outcomes included epithelial healing time, visual acuity, pain relief, recurrence rates, and complication profiles. Abbreviations: AMT: amniotic membrane transplantation, AMT group: patients treated with AMT, AMG: amniotic membrane graft, BCL: bandage contact lens, CF: conjunctival flap, M: male, F: female, PED: persistent epithelial defect, RCT: randomized controlled trial, UCVA: uncorrected visual acuity, VA: visual acuity, vs: versus, ↓: decreased, ↑: increased

Author, Year	Study design	Country	Sample size	Participant characteristics	Intervention details	Comparator details	Outcomes	Results
Khokhar et al., 2005 [1]	Randomized controlled trial	India	30 eyes (15 AMT, 15 control)	Age: 37 ± 14.7 yrs; Gender: 16M/14F; Neurotrophic ulcers	Single/multi-layer AMT depending on ulcer depth	Tarsorrhaphy or BCL	Healing time, VA improvement, and success rate	Healing: 73.3% AMT vs 66.7% control; VA improved: 47% vs 33%; median healing time: 21 days
Lee & Tseng, 1997 [6]	Prospective case series	USA	11 eyes (1 per patient)	Age: 69.9 ± 16.3 yrs; Gender: 6M/5F	Cryopreserved AMT, 1–3 layers, sutured; bandage lens + Maxitrol	None	Epithelial healing, time to healing, recurrence	10/11 healed in 3.9 ± 2.3 wks (vs 17.5 ± 13.9 wks pre-AMT); no recurrence in 9 ± 5.9 months
Brijačak et al., 2008 [7]	Prospective interventional	Croatia	18 patients	Age: 54 ± 17 yrs; Gender: 11M/7F	AMT over cornea/conjunctiva; IL-1α and IL-1ra assessed	None	Epithelial healing, visual acuity improvement	89% success (16.4 days); VA improved in 16/18; no major complications over 17 months
Abdulhalim et al., 2014 [8]	Randomized clinical trial	Egypt	40 eyes (20 CF, 20 AMG)	CF: 52.75 ± 12.03 yrs, 65% M / 35% F; AMG: 47.8 ± 15.87 yrs, 45% M / 55% F	Bipedicle conjunctival flap or cryopreserved AMG	CF vs AMG	Epithelialization time, infection control, visual acuity, complications	Success rate 90% in both groups; no significant difference in healing or VA (p > 0.75)
Linhares et al., 2024 [9]	Prospective case series	Brazil	11 eyes of 11 patients	Age: Median 60 (range 34–82); Gender: 64% M, 36% F	Cryopreserved AMT for refractory neurotrophic ulcers	None	Visual acuity, pain relief, epithelial closure, complications	VA improved in 36%, worsened in 18%; pain relieved in 66%; epithelial closure in 54.6%; failure rate 45.5%
Kheirkhah et al., 2012 [10]	Prospective case-control	Iran	25 eyes (14 AMT, 11 control)	Age: 25.1 ± 6.6 yrs; Gender: 21F/4M; Contact lens users	Topical antibiotics + single-layer AMT, overlay patch technique	Topical antibiotics only	Pain score, healing time, VA, opacity, neovascularization	Pain ↓ (p=0.001); healing: 13.2±2.6 vs 15.5±3.4 days; UCVA better (p=0.03); opacity ↓ (p=0.04)
Prabhasawat et al., 2001 [11]	Prospective interventional	Thailand	28 eyes (Group A:10, B:13, C:5)	Most common: neurotrophic keratopathy	Group A: single-layer AMT; Groups B/C: multilayer AMT	Internal group comparison	Epithelial healing, stromal thickness, vision, and failure rate	82.1% total success; faster healing with multilayer (p=0.014); stromal thickness ↑ (p=0.022); VA improved in 28.6%
Trac et al., 2016 [5]	RCT	Iran	60 eyes (30 AMT, 30 control)	Corneal ulcers of various etiologies	Cryopreserved AMT	Conventional therapy	Healing rate, healing time, and VA improvement	Healing: 93.3% AMT vs 76.7% control (p<0.05); faster healing (p=0.03); VA better in AMT group
Ma et al., 2002 [12]	Prospective interventional	USA	23 eyes	PED, neurotrophic, or exposure keratopathy	Single or multilayer AMT, depending on ulcer severity	None	Epithelial closure, recurrence, complications	Closure in 87% (20/23); no recurrence over 6 months

Etiologies included infectious keratitis (fungal, bacterial, or Acanthamoeba), neurotrophic ulcers, exposure keratopathy, and post-surgical epithelial defects. Neurotrophic ulcers were the primary focus in some studies [1,9], while infectious causes predominated in others [8,12]. AMT was applied either alone or alongside conventional treatments. Surgical approaches varied from single-layer to multilayer AMT, especially for deeper ulcers or perforations [1,11]. Comparators included conjunctival flaps [8], bandage contact lenses, and medical therapy alone [1,10,12]. Follow-up duration ranged from 1 to 37 months, commonly 3 to 6 months.

Quality Assessment

The three randomized controlled trials scored highest on quality assessment, with one scoring 25/27 on the Modified Downs and Black checklist [5]. Observational studies scored between 20 and 22, though some lacked detailed reporting on randomization or allocation concealment [6,9,10]. The lowest scores (17-19) were found in early or descriptive case series [13], mainly due to limited control for confounding and the absence of comparator groups (Table 2).

**Table 2:** Methodological quality scores of selected studies on amniotic membrane transplantation for corneal ulcers and persistent epithelial defects This table presents the methodological quality assessment of selected studies investigating amniotic membrane transplantation in the treatment of corneal ulcers and persistent epithelial defects. Quality was evaluated across five domains: reporting, external validity, internal validity related to bias and confounding, and statistical power. Scores reflect adherence to methodological standards, with a maximum total score of 27. Abbreviations: AMT: amniotic membrane transplantation; PED: persistent epithelial defect

Author, Year	Reporting (10)	External Validity (3)	Internal Validity – Bias (7)	Internal Validity – Confounding (6)	Power (1)	Total Score (27)
Khokhar et al., 2005 [1]	9	3	6	5	1	24
Lee & Tseng, 1997 [6]	9	2	5	3	1	20
Brijačak et al., 2008 [7]	8	2	4	2	1	17
Abdulhalim et al., 2014 [8]	9	3	6	5	1	24
Linhares et al., 2024 [9]	9	2	5	3	1	20
Kheirkhah et al., 2012 [10]	9	2	6	4	1	22
Prabhasawat et al., 2001 [11]	9	3	6	4	1	23
Ma et al., 2002 [12]	8	2	5	3	1	19

Overall, the reporting quality was high, with clear objectives, defined outcomes, and detailed descriptions of interventions. External validity was moderate, reflecting tertiary center settings. Blinding was largely unfeasible due to the surgical nature of the intervention, but outcome assessments were generally appropriate and clearly defined. Statistical power was adequate across studies.

Effect on Epithelial Healing Rates

AMT was associated with high rates of corneal epithelialization. Complete re-epithelialization was reported in 90.9% of cases, with mean healing times significantly shorter than those in the pre-AMT period [6]. Other studies reported healing success rates of 89% [7], 93.3% compared to 76.7% in controls (p < 0.05) [12], and an overall rate of 82.1%, with faster healing in multilayer AMT cases [11]. Lower healing rates of 54.4% were noted in refractory neurotrophic ulcers [9].

Effect on Time to Epithelialization

Several studies noted faster healing times with AMT. Mean healing times ranged from 13.2 ± 2.6 days with AMT versus 15.5 ± 3.4 days in controls (p = 0.07) [10], with statistically significantly faster epithelialization in some cohorts (p = 0.03) [12]. Multilayer AMT yielded significantly faster healing compared to single-layer applications (p = 0.014) [11]. Median healing time in neurotrophic ulcers was approximately 21 days with AMT, comparable to controls [1].

Effect on Visual Acuity Outcomes and Pain Reduction

Visual acuity (VA) improvement following AMT varied across studies. VA improved in 47% of AMT cases versus 33% in controls [1] and in 16 of 18 cases [7]. Lower improvements were observed in some studies (36%), with 18% experiencing deterioration [9]. Statistically significant gains in best-corrected and uncorrected VA were reported in other cohorts [10,12]. Pain relief was documented with substantial decreases in pain scores post-AMT (e.g., from 2.4 ± 0.5 to 1.1 ± 0.9; p = 0.001) [10], with 66% of patients experiencing complete symptom relief in some cohorts [9].

Effect on Complications and Recurrence

Complications were minimal. No serious adverse events were reported in several studies with follow-up up to 26.7 months [5,13]. Amniotic membrane dissolution occurred in some cases but did not prevent re-epithelialization [5]. One corneal perforation was reported post-AMT, managed conservatively [1]. Multilayer AMT prevented perforation and preserved corneal integrity in stromal thinning cases [11].

Discussion

This systematic review demonstrates that AMT is an effective adjunctive treatment for corneal ulcers, enhancing epithelial healing, reducing pain, and improving visual outcomes. From an initial 2,287 records screened across multiple databases, nine studies met the inclusion criteria, encompassing diverse patient populations and AMT techniques. The evidence consistently showed that AMT accelerates epithelial closure compared to standard therapies, likely due to its anti-inflammatory and anti-scarring effects. Studies reported up to 30% faster healing rates and reductions in average healing time by several days [13]. Moreover, AMT provided significant pain relief, probably through protection of nerve endings and inflammation modulation, with pain scores dropping markedly within 48 hours post-application [11-13]. Visual acuity improvements were noted, especially in patients with central corneal ulcers, although the degree of recovery varied according to ulcer severity and location [2-4,6-8].

These findings support the clinical utility of AMT as a valuable therapeutic option in managing persistent and refractory corneal ulcers. The broad consistency of outcomes across randomized trials and observational studies reinforces its role in enhancing corneal epithelialization and patient comfort. However, variability in ulcer etiology, AMT application techniques, and follow-up duration highlights the need for further large-scale, standardized trials to optimize protocols and confirm long-term benefits. Nonetheless, the current evidence base underscores AMT’s potential to improve healing and visual prognosis in this challenging clinical context.

While AMT demonstrates clear benefits in accelerating epithelial healing and reducing pain in corneal ulcers, especially neurotrophic ulcers, its impact on long-term visual acuity improvement is less pronounced. This may be attributed to the biological process following AMT, where the transplanted amniotic membrane is gradually reabsorbed and replaced by newly formed fibrotic stromal tissue. Such fibrotic remodeling can reduce corneal transparency compared to the native corneal stroma, potentially limiting visual recovery despite successful epithelialization. This phenomenon underscores the need to balance epithelial healing with stromal clarity when evaluating the overall efficacy of AMT in corneal ulcer management. Future studies should further investigate techniques to mitigate stromal fibrosis post-AMT to optimize visual outcomes.

Limitations

This review faced several limitations, including heterogeneity in patient populations, AMT techniques, and outcome measures, which complicated data synthesis. Most studies had small sample sizes, limiting generalizability, and varied follow-up durations, restricting the evaluation of long-term efficacy and safety. Publication bias is possible, as positive results are more likely to be published, and excluding non-English studies may have omitted relevant data.

## Conclusions

This systematic review highlights the potential of AMT as a valuable adjunctive treatment for corneal ulcers and persistent epithelial defects. The included studies demonstrate that AMT can facilitate epithelial healing, reduce ocular pain, and, in some cases, improve visual acuity. These therapeutic effects are consistent with the known biological properties of the amniotic membrane, including its anti-inflammatory, anti-fibrotic, and epithelial-promoting functions.

However, the current evidence base is limited by methodological weaknesses, including small sample sizes, inconsistent outcome reporting, and heterogeneity in both patient populations and AMT techniques. While findings are encouraging, the lack of standardized protocols and long-term follow-up limits the ability to draw firm conclusions regarding efficacy and generalizability. High-quality, randomized controlled trials with uniform criteria and longer follow-up are needed to clarify the optimal use of AMT and to support its integration into standardized clinical practice.
